# Single cell transcriptomics reveals dendritic cell subsets in bovine afferent lymph and immune cell-resolved responses to BCG vaccination

**DOI:** 10.3389/fimmu.2026.1764014

**Published:** 2026-04-07

**Authors:** Rachrapee Sukmak, Heather A. Mathie, Richard S. Taylor, Jianxuan Sun, Barbara Shih, Charlotte R. Bell, Mark Gray, Daniel J. Macqueen, Jayne C. Hope

**Affiliations:** 1 The Roslin Institute and Royal (Dick) School of Veterinary Studies, The University of Edinburgh, Midlothian, United Kingdom; 2 School of Infection and Immunity, College of Medical, Veterinary and Life Sciences, University of Glasgow, Glasgow, United Kingdom; 3 Department of Biomedical and Life Sciences, Lancaster University, Lancaster, United Kingdom

**Keywords:** antigen presenting cells, Bacille Calmette-Guérin vaccine, bovine immunology, dendritic cells, single cell RNA-seq

## Abstract

Bovine tuberculosis (bTB), caused by *Mycobacterium bovis* (*M. bovis*), remains an ongoing global issue for human and animal health. The Bacille Calmette Guerin (BCG) vaccine offers immunity against bTB, however, the mechanisms underlying the heterogenous protective response, including variations across species and age groups requires further investigation. In this study, we focused on dendritic cells (DCs), which are crucial for adaptive immune stimulation following BCG vaccination. By capturing afferent lymph DCs (ALDCs) migrating from the skin, we investigated shifts in DC profiles and potential subset-specific functions in response to BCG vaccination. Single-cell RNA sequencing (scRNA-seq) was performed on samples from *Bos taurus* calves (n=3) before and after BCG vaccination, capturing the transcriptome of 20,761 individual cells expressing on average 3,036 genes, which were clustered into ALDCs, monocytes, T-cells, B-cells and NK cells. The ALDC subsets were further identified as cDC1 and cDC2. In homeostasis, ALDCs expressing potential subset-specific genes for cDC1, including ENSBTAG00000056208, *STX4*, *NEBL*, *ADAM23*, *ART3*, and cDC2; *FN1*, *PSPH*, *FGL2*, *SHOX2*, and *WWTR1* were identified. Following BCG vaccination, while both DC subsets exhibited gene expression signatures indicative of antigen-presenting function, migration, and DC maturation, cDC1 showed upregulation of genes consistent with metabolic alterations and lymphocyte recruitment, whereas cDC2 upregulated genes consistent with inflammatory responses. Overall, this study comprehensively describes the transcriptomic landscape of bovine ALDC subsets, providing evidence for the importance of subset-specific genes to BCG vaccination responses, while advancing knowledge on how ALDCs contribute to protective immunity against bTB.

## 1 Introduction

Bovine tuberculosis (bTB) is a zoonotic disease that negatively impacts animal health, with major global economic costs. The causative pathogen, *Mycobacterium bovis* (*M. bovis*), can be transmitted across wildlife, livestock, and humans (1). The nature of Mycobacteria, including the ability to reside in intracellular locations and evade host immunity, leads to infections often being undetected and subclinical (2). Without timely diagnosis, *M. bovis* infection typically progresses in the lung of cattle and may circulate throughout herds, leading to outbreaks of bTB. Vaccination alongside efficient testing is necessary for disease eradication and achieving herd immunity. In the UK, multiple regions continue to report bTB cases, with control measures costing approximately £100 million per annum (3). In 2021, the Bacillus Calmette-Guérin (BCG) vaccine was approved for field trials in cattle, to mitigate the disease along with development of Differentiating Infected from Vaccinated Animals (DIVA) diagnostic tests (4).

BCG is an attenuated live bacterial vaccine that has been used for over a century (5). Despite this, the immunological mechanisms underlying its protective actions are unresolved in both cattle and humans (6, 7), including the variation in protective immunity (e.g., age-dependent effects). In calves, BCG offers protective immunity lasting up to 12 months, but not extending to 24 months (8), which can be boosted by further vaccination (9). In humans, BCG induces long-term protection, ranging from 10–20 years (10) to 50–60 years (11), with more favourable outcomes when children are vaccinated (12). Recent hypotheses suggest protective responses induced by BCG are also associated with epigenetic modification and metabolic reprogramming of the innate immune system, so-called ‘trained immunity’, providing non-specific protection against heterogenous pathogens. Further investigation into the immune response to BCG is needed to provide a comprehensive understanding of the vaccine, improve its effectiveness, and support the development of next-generation tuberculosis vaccines.

Tuberculosis vaccine efficacy depends on the ability to induce cell-mediated immune responses mainly through interferon-gamma (IFN-γ)-induced activation of intracellular killing mechanisms, as well as cytotoxic mechanisms to contain and eliminate infected cells. Dendritic cells (DCs), macrophages, and monocytes recognise *M. bovis* via pattern recognition receptors (PRR), such as toll-like receptors (TLRs) and intracellular nucleotide oligomerization domain-like receptors, leading to pro-inflammatory cytokine responses (13, 14). Antigen presentation by infected cells promotes CD4+ T-cells to secrete IFN-γ, while antigen recognition by CD8+ T-cells induces cytotoxic activity to remove infected cells (15). NK cells and neutrophils are also recruited to the infection site through chemokines produced by infected macrophages (16). NK cells participate in *M. bovis* clearance by releasing perforin and granulysin, while enhancing immune responses via IFN-γ secretion (6).

The induction of protective immune responses by vaccination requires the activity of DCs. DCs are professional antigen-presenting cells (APCs) with key roles bridging innate and adaptive immunity. They act as sentinels residing in the skin and mucosal surfaces and recognise pathogens or vaccines entering the body. Following recognition through PRRs, DCs uptake and process antigens that are then loaded onto major histocompatibility complex (MHC) class I and II molecules, and presented to CD4+ and CD8+ T-cells, respectively. DCs comprise subsets with distinct phenotypes and specialised functions, including conventional DC type 1 (cDC1), specialised in cross-presentation of intracellular pathogen antigens to CD8+ T-cells, and cDC2, with both immune enhancing and regulatory functions (17). Following BCG vaccination in cattle, DCs at the injection site migrate towards the draining lymph node, where T-cell activation and immune induction takes place (18).

In this study, we performed bovine afferent lymphatic cannulation in order to collect and analyse DCs draining from the site of vaccination *ex-vivo* (19). This model allows trafficking afferent lymph DCs (ALDCs) to be captured in both the steady state, and following vaccination. This model can provide insights into how DCs respond and communicate after BCG encounter, contributing to protective responses. Examining functional, phenotypic and gene expression profiles of DCs can provide unique insights into the immunological mechanisms induced by BCG vaccination that can lead to protective immune induction. By using high forward scatter (FSc) and high side scatter (SSc) as a sorting strategy, cells that displayed DC characteristics (20) were separated from other immune cell populations within afferent lymph as enriched DC samples. This strategy has been used in previous studies to identify DCs (19, 21).

Single-cell RNA sequencing (scRNA-seq) is a transformative technology for profiling global gene expression at the single-cell level (22). scRNA-seq has been applied to a variety of organ systems and species, aiding the discovery of functional cellular diversity within the immune system, capturing immune cell subpopulations, differentiation states, and functional responses to pathogens (23). Recent work characterised and investigated APCs, DCs and monocytes in cattle mesenteric lymph nodes, reporting transcriptomic profiles for cDC1, cDC2, migratory dendritic cells (miDC), resident DCs (resDC), progenitor DCs, plasmacytoid DCs (pDC), and putative dendritic cell type 3 (DC3) (24). Such work provides a reference that should be expanded following immune system activation to reveal more defined or specialised immune subpopulations and their potential functions.

Immune responses to BCG vaccination have been studied using scRNA-seq in diverse species. A study examining the effects of BCG vaccination against COVID-19 in hamsters showed that BCG reduced lung viral load, increased T-cell recruitment, and shifted T-cell transcriptional profiles towards antigen processing (25). An analysis of human peripheral blood mononuclear cells revealed that BCG increased effectiveness of innate immune responses, confirming the essential role of IFN-γ upregulation in high responder patients. The same authors identified *STAT1* as essential for trained immunity in monocytes (26). Another scRNA-seq study of human monocytes indicated that BCG trains innate immune cells, influencing interactions between APCs and T-cells, altering response levels to LPS, and activating the expression of master immune regulators (27).

The above examples show that scRNA-seq has the potential to transform our understanding of protective immunity to BCG vaccination. However, this technology is yet to be applied to study the responses of the cattle immune system to BCG. Therefore, this investigation leveraged scRNA-seq to identify immune cell subpopulations in the afferent lymph of cattle, with the specific aim of establishing the early transcriptomic response of ALDC subsets to BCG vaccination. Past work has shown that the first 24 hours post-vaccination is a crucial period for BCG uptake and carriage by ALDCs (28), affecting antigen presentation and the induction of protective immunity against bTB. Comparing enriched populations of ALDCs sampled before and 24 hours post-vaccination, we investigate the presence of DC subsets that differ at homeostasis, before uncovering their shared and differential responses to BCG vaccination.

## 2 Materials and methods

### 2.1 Animal usage and sample collection

**Figure 1:**
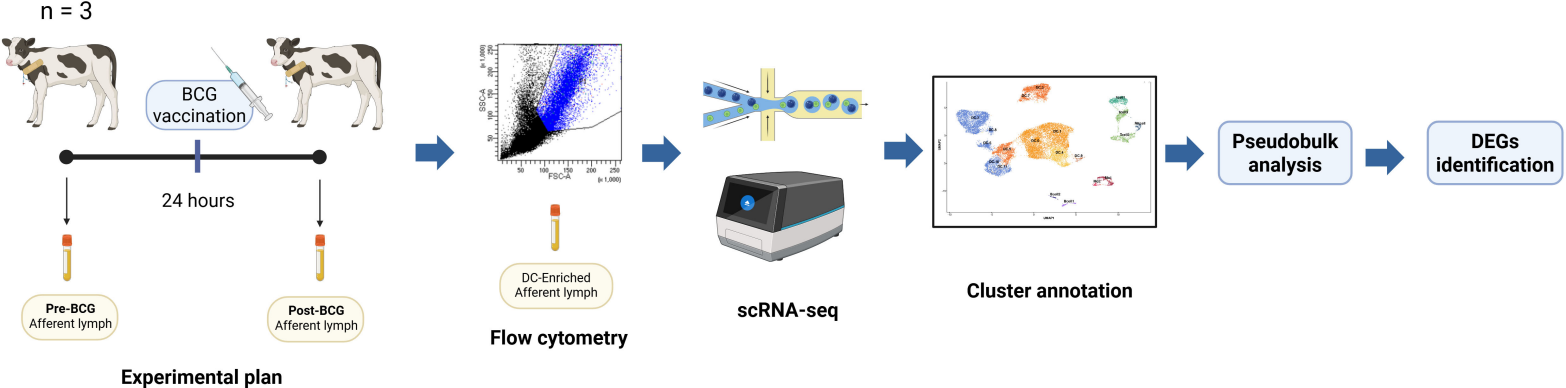
Summary of experimental design. Afferent lymph was collected from three calves prior to BCG vaccination (pre-BCG) and 24 hours after BCG vaccination (post-BCG), followed by flow cytometry for ALDC-enriched afferent lymph, scRNA-seq, and data analysis. The image was created using BioRender.com.

Three *Bos taurus* calves (two male Holstein-Friesian [calves 1 and 2], and one male Norwegian Red [calf 3]), aged 3–6 months and sourced from TB-free farms (University of Edinburgh Langhill Farm, Roslin, Midlothian, UK), underwent surgery for the cannulation of a pseudo-afferent superficial cervical lymph vessel, as described in a previous study (19). Prior to BCG vaccination (‘pre-BCG’), samples were collected at 6–7 days post-cannulation (DPC). The BCG strain Danish (SSI, Denmark) was administered via the subcutaneous route (approximately 1 x 10^6^ bacteria, equivalent to 5x human dose) followed by sample collection at 24 hours after BCG vaccination (‘post-BCG’) (**Figure 1**). Afferent lymph collected from the two timepoints, pre-BCG and post-BCG, was cryopreserved in foetal bovine serum with 10% dimethyl sulfoxide (DMSO) at -155 °C (20) prior to cell sorting. All experimental protocols were approved in accordance with the UK Animals (Scientific Procedures) Act, 1986, (PPL: 60/4394) and The Roslin Institute’s Local Animal Welfare and Ethical Review Board.

### 2.2 Cell preparation and scRNA-seq library generation

Cells from afferent lymph were thawed and washed twice with PBS and then centrifuged at 400*g* for 5 minutes. Cells were resuspended and stained with Zombie aqua (Biolegend) to assess cell viability. After being incubated with Zombie aqua for 15 minutes in the dark at room temperature, the washing step was repeated. Cells were resuspended with 1 mL of RPMI medium then placed through a cell strainer (70 µm, Falcon™) and placed on ice.

DCs were enriched using Fluorescence-Activated Cell Sorting (FACS) to sort cells based on viability, high FSc and high SSc properties. The gating strategy - targeting cells with large size and high granularity - was based on previous studies that showed these properties strongly enrich for DCs (21, 29). Following gating, an average 14.02% of the total afferent lymph cells appeared in the high FSc/SSc gate, matching expectations for ALDC proportions (19, 20).

The average cell viability across all six samples following cell sorting was 91.83%. A total of 1 x 10^5^ sorted cells were utilised for scRNA-seq library preparation per sample, performed at the Queen’s Medical Research Institute (QMRI). Approximately, 20,000 cells were loaded aiming for ~10,000 recovered cells per sample. Libraries were generated according to the 10x Genomics Single Cell v3 Reagent Kit protocol with 12 PCR cycles, followed by cDNA concentration measurement by Qubit. Each library was tagged with individual i7 indexes and underwent quality control using a Bioanalyser High Sensitivity Chip. Sequencing was performed on an S2 flow cell on the Illumina NovaSeq 6000 sequencing platform at Edinburgh Genomics.

### 2.3 scRNA-seq quality control and clustering

scRNA-seq FASTQ files were aligned to the bovine reference genome (*Bos taurus*; ARS-UCD1.3, GCA_002263795.3) using Cell Ranger v8.0.1 (30). The ‘mkref’ function was used to generate the bovine index genome, and the ‘count’ function was employed for genome alignment. Additionally, ambient RNA in each sample file was removed using CellBender v0.3.2 (31), with the parameters ‘--expected-cells’ and ‘--total-droplets-included’ configured according to the barcode rank plot of individual samples, acquired from the Cell Ranger count report. Seurat v5.1.0 (32) was utilised for subsequent downstream analysis. Cells containing gene counts < 200 and > 10% percent mitochondrial genes were excluded. DoubletFinder v2.0.4 (33) was applied to identify and remove potential doublets from the dataset.

Following quality control, all samples were merged. The pre-processing steps were conducted, including normalisation with the ‘LogNormalize’ method, finding variable genes, and scaling data. Principal component analysis (PCA) was employed for dimensionality reduction, alongside the ‘ElbowPlot’ function to determine an appropriate number of principal components (30 principal components). Data integration with batch correction was performed using the ‘IntegrateLayers’ function with the ‘HarmonyIntegration’ method (34). To establish cluster resolution, the clustree v0.5.1 (35) and SC3 v1.34.0 (36) packages were utilised, with selected resolution based on the number and stability of clusters. Subsequently, clustering was carried using the ‘FindNeighbors’ and ‘FindClusters’ functions with resolution values of 1.2.

### 2.4 Cluster annotation and subset annotation

Marker genes for each cluster were identified using the ‘FindAllMarkers’ function in Seurat with adjusted P-value < 0.05 and average log2FC > 0.5. Clusters were initially annotated based on known marker genes of bovine immune cells. Specifically, *FLT3* was used as a marker for DCs (37), *CSFR1* and *FCGR3A* (CD16) for monocytes (38), *CD79B* and *JCHAIN* for B-cells (39), *CD3E* for T-cells (40), and *NCR1* for NK cells (41). Manual doublet removal was subsequently performed by focusing on cells demonstrating mixed expression of these marker genes, such as cells expressing both *FLT3* (DCs) and *FCGR3A* (monocytes), indicating potential doublets. Five cell types were identified as core populations, including DCs, monocytes, B-cells, T-cells, and NK cells. Subsets within core populations were then annotated using the following marker genes, which were differentially expressed in the individual clusters, including *DPP4* (CD26) or *CADM1* for cDC1 (24, 28, 42); *SIRPA* or *TLR1* for cDC2 (28, 43); *CSF1R* and *CD14* for classical monocytes (cM) (37, 38, 42, 44); *CSF1R*, *CD14*, and *FCGR3A* for intermediate monocytes (intM) (37, 38, 42, 44); *CD79A*, *CD79B*, *JCHAIN*, *EBF1*, and *PAX5* for putative pre B-cells (39, 45, 46); *CD79A*, *CD79B*, and *JCHAIN* for putative mature B-cells (39, 45); *CD3E* and *WC1* for WC1+ γδ T-cells (47, 48); *CD3E* and *CD4* for helper T-cells (Th cells) (24, 49); *NCR1* for NK cells (18, 50) (**Supplementary Table 1**).

### 2.5 Pseudo-bulk and differential gene expression analysis

To identify differentially expressed genes (DEGs) following BCG vaccination in cell types of interest, pseudo-bulk analysis was performed on the global Seurat object using the ‘AggregateExpression’ function. This approach reduces false positives by appropriately accounting for variation among biological replicates (51, 52), aggregating gene counts based on individual cell types, individual animals, and experimental conditions (here: pre- and post-BCG vaccination). DEGs were identified using the ‘FindMarkers’ function, with ‘DESeq2’ as the testing method. The results were visualised by volcano plots generated using the ‘EnhancedVolcano v1.13.2’ (53) package (adjusted P-value < 0.05 cut-off). All DEGs (i.e., those both upregulated and downregulated post-BCG), were compared across all cell types using an upset plot generated by the UpSetR v1.4.0 package (54). Furthermore, we conducted differential gene expression analysis comparing cDC1 and cDC2 at homeostasis (i.e., pre-BCG) using ‘FindMarkers’ function with adjusted P-value < 0.05 as a cut-off.

## 3 Results

### 3.1 scRNA-seq identifies immune cell populations in bovine afferent lymph

**Figure 2:**
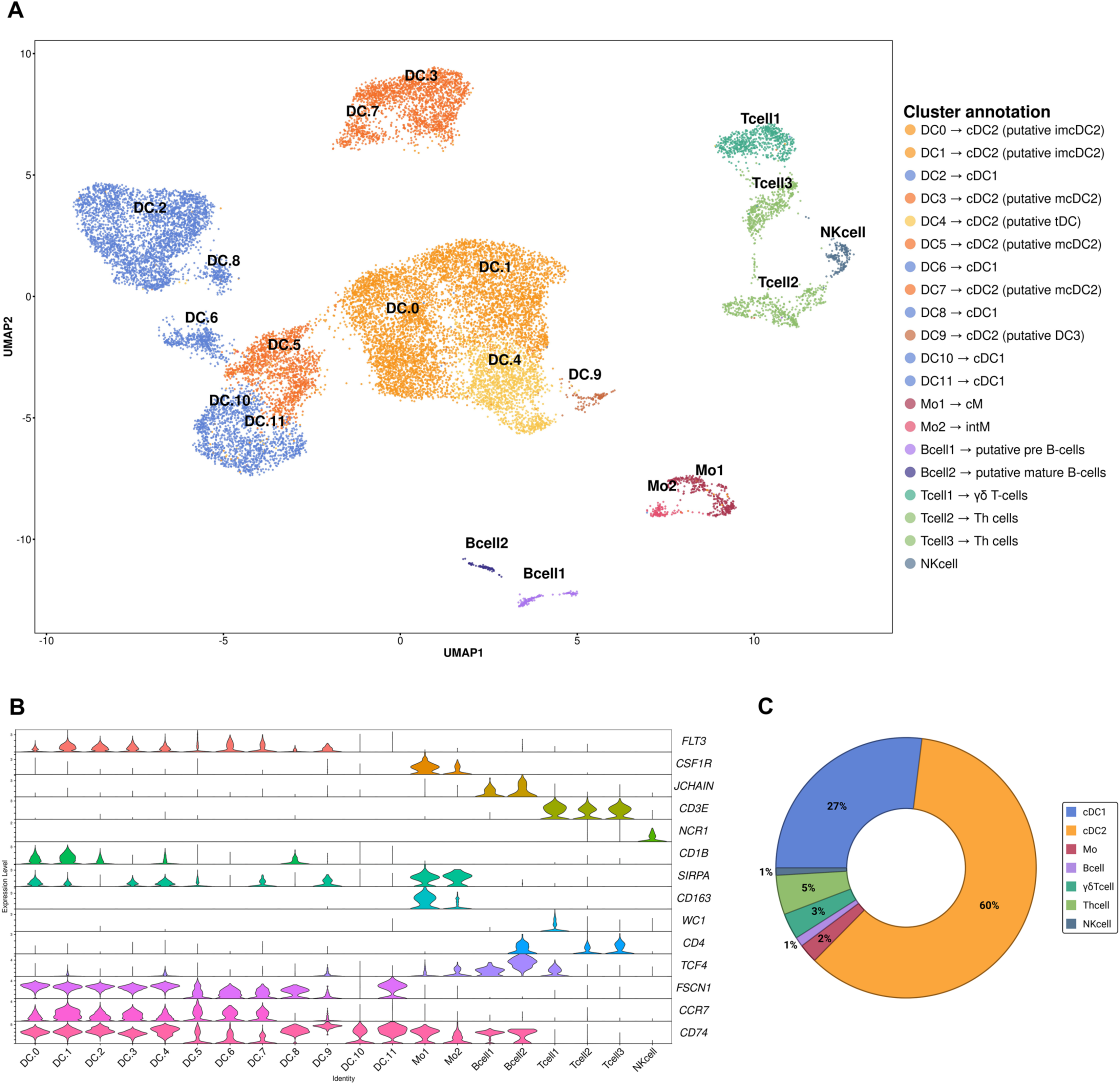
**(A)** UMAP showing 20 clusters with legend indicating putative identities of each cell population. **(B)** Violin plots displaying the expression of marker genes used for putative immune cell subset annotation. **(C)** Donut chart exhibiting the proportion of major cell populations captured from all samples combined together, with no separation by vaccine conditions.

Six samples were used in scRNA-seq, one taken pre-BCG vaccination and one 24 hours post-BCG vaccination for each sampled calf (i.e., n=3 pre-BCG; n=3 post-BCG). Across all samples, we obtained 20,761 high quality cells and detected the expression of 3,036 genes per cell on average (**Supplementary Table 2**). On average, 32.5% of cells were excluded during quality control steps (see Methods). Correlation analysis and PCA using raw gene counts for all six samples was performed to explore variation across the three sampled calves, revealing no impact of breed, and high reproducibility in all samples (**Supplementary Figure 1**). The cells were then clustered and annotated using *a priori* defined marker genes. This strategy identified five core cell types; DCs (including cDC1 and cDC2), monocytes, B-cells, T-cells, and NK cells across 20 distinct clusters (**Figure 2A**; **Supplementary Figure 2**) including putative subsets (**Figure 2B**). DC populations were initially collapsed to fewer clusters within the global dataset to avoid over clustering due to the high heterogeneity observed within this population (**Supplementary Figures 3A, B**). As expected, the captured cells were dominated by DCs (cDC1 and cDC2: 27% and 60% of all cells, respectively), with a much smaller proportion annotated as T-cells (Th cells and WC1+ γδ T-cells: 5% and 3% of all cells, respectively), monocytes (2% of all cells), B-cells (1% of all cells), and NK cells (1% of all cells) (**Figure 2C**). All DEGs and the top marker genes identified in each cluster are presented in **Supplementary Table 3** and **Supplementary Figure 4**. The ALDC populations were consistently represented among the three calves both pre- and post-BCG (**Supplementary Figure 5**).

### 3.2 Identification of ALDC subsets and non-DC immune subpopulations

**Figure 3:**
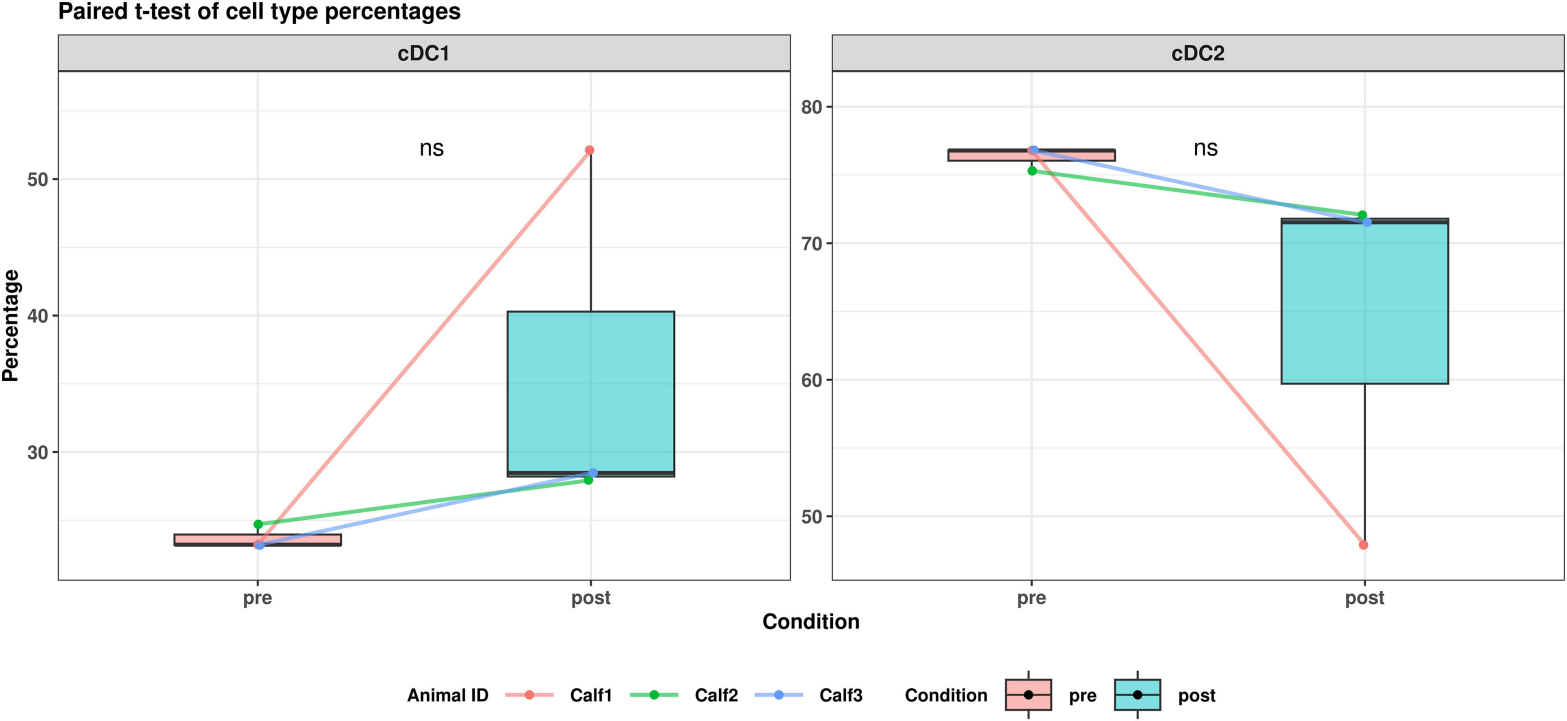
Bar charts for pre- and post-BCG conditions using the normalised percentage of cDC1 and cDC2, with individual lines showing the same data from the individual animals. A paired T-test showed no significant changes between conditions in both subsets.

ALDC clusters were divided into putative cDC1 (clusters DC2, DC6, DC8, DC10, DC11) and cDC2 (clusters DC0, DC1, DC3, DC4, DC5, DC7, DC9) subsets. cDC1 *vs*. cDC2 annotations were mainly distinguished by *SIRPA* expression in cDC2 (**Supplementary Figure 6**). Within the cDC2 population, four putative subsets were annotated and named immature cDC2 (imcDC2), mature cDC2 (mcDC2), transitional DC (tDC), and DC3. Two subsets were annotated according to their *CD1B* expression, with expression of *CD1B* indicating imcDC2 (cluster DC0, DC1), and remaining clusters annotated as mcDC2 (cluster DC3, DC5, DC7) (37). The putative tDC (cluster DC4) and DC3 (cluster DC9) subsets were annotated based on the DEGs in each cluster; tDC: *SPIB, BLNK, IL3RA* (**Supplementary Figure 7**), and DC3: *FCER1G, XDH, F2RL2, CKB, VIM, S100A11* (55, 56) (**Supplementary Figure 8**). Additionally, *FSCN1* and *CCR7* (37) were expressed across all ALDC clusters, consistent with their migratory profile (**Supplementary Figure 9**), alongside genes related to antigen presentation, i.e., *CD74*, *HLA-DRA*, and *LY75* (24) (**Supplementary Figure 10**). *BATF3*, typically used to differentiate cDC1 and cDC2 (42) was expressed in the majority of ALDCs (**Supplementary Figure 11**). We observed no change in the proportion of ALDC populations between conditions (**Figure 3**).

Although, the identified non-DC populations were not included in further analyses, we briefly describe them and share their marker genes to aid future studies in the field. The monocyte population was identified based on expression of *CSF1R* and further divided into intM (Mo1; *CD14* and *FCGR3A*) and cM (Mo2; *CD14*). Among three T-cell clusters, we identified WC1+ γδ T-cells (T1) according to significant upregulation of *WC1*, while T2 and T3 expressed *CD4* and were characterised as Th cells. B1, expressing *EBF1* and *PAX5*, potentially represent pre B-cells (46, 57). In contrast, absence of both genes as significant marker genes in B2 may indicate an active or mature B-cell population.

### 3.3 Transcriptomic profiles of homeostatic ALDCs

The transcriptomes of cDC1 and cDC2 were compared at homeostasis (i.e., pre-BCG) to reveal subset-specific gene expression using a pseudo-bulk approach. As a result, cDC1 showed 144 DEGs, with the top-five upregulated as follows: ENSBTAG00000056208 potentially encoding long interspersed nuclear elements (LINE-1) retrotransposable element ORF2 protein isoform X1, syntaxin 4 (*STX4*), nebulette (*NEBL*), ADAM metallopeptidase domain 23 (*ADAM23*), and ADP-ribosyltransferase 3 (*ART3*) (**Supplementary Table 4**). cDC2 showed 202 DEGs with fibronectin 1 (*FN1*), phosphoserine phosphatase (*PSPH*), fibrinogen like 2 (*FGL2*), SHOX homeobox 2 (*SHOX2*), and WW domain containing transcription regulator 1 (*WWTR1*) as the top-five upregulated (**Supplementary Table 5**).

### 3.4 Changes in ALDC transcriptome following BCG vaccination

**Figure 4:**
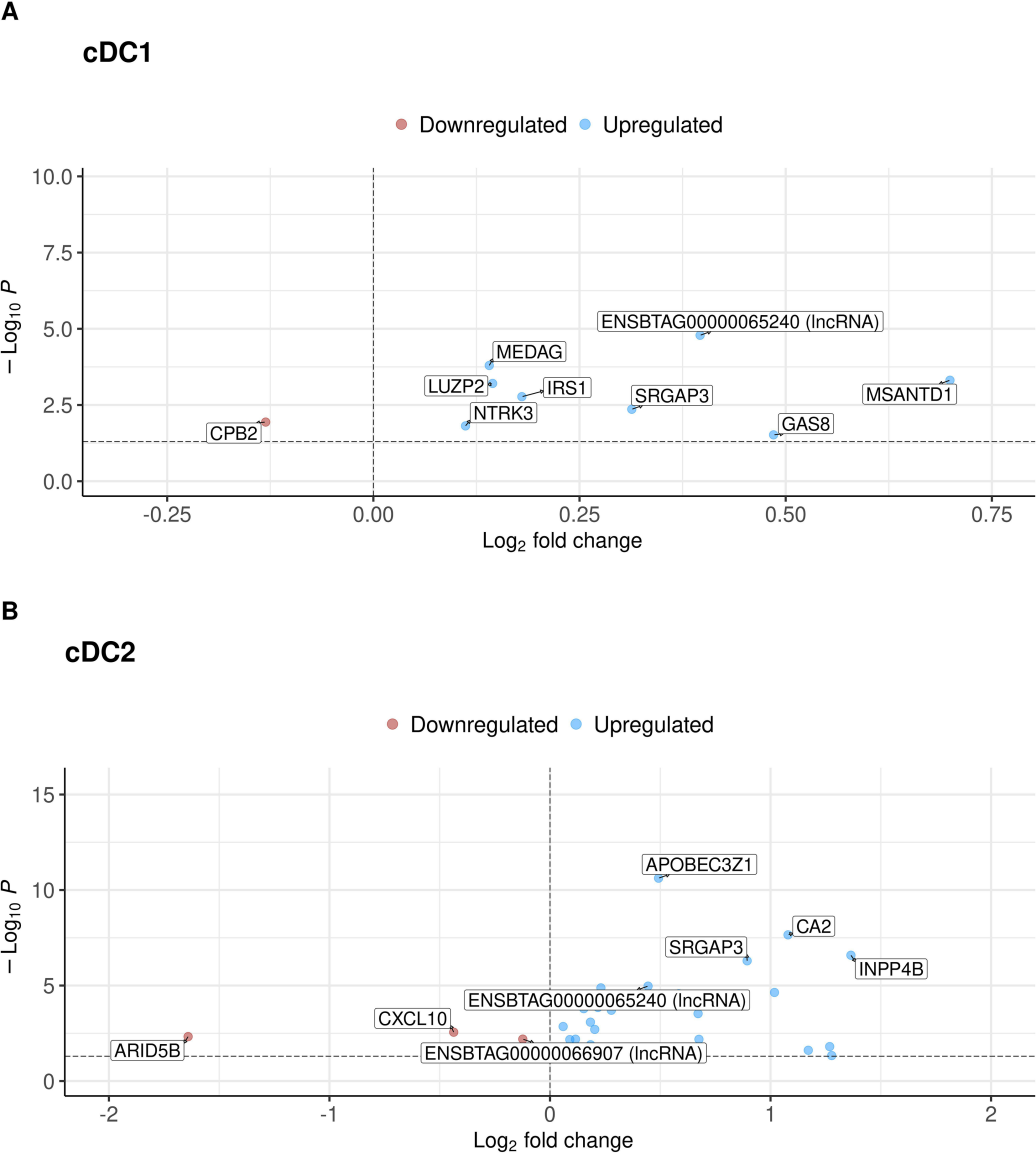
Volcano plots showing DEGs comparing pre- and post-BCG for cDC1 **(A)** and cDC2 **(B)**, using a pseudo-bulk approach. Negative and positive log2 fold change values indicate downregulation and upregulation after BCG vaccination, respectively.

Compared to the pre-BCG state, post-BCG cDC1 showed nine DEGs, with only one, carboxypeptidase B2 (*CPB2*), downregulated. The other upregulated genes included ENSBTAG00000065240 (a long non-coding RNA [lncRNA]), mesenteric estrogen dependent adiposis (*MEDAG*), Myb/SANT DNA binding domain containing 1 (*MSANTD1*), leucine zipper protein 2 (*LUZP2*), insulin receptor substrate 1 (*IRS1*), SLIT-ROBO Rho GTPase activating protein 3 (*SRGAP3*), neurotrophic receptor tyrosine kinase 3 (*NTRK3*), and growth arrest specific 8 (*GAS8*) (**Figure 4A**; **Supplementary Table 6**).

In cDC2, twenty-nine DEGs were identified following BCG vaccination, with twenty-six upregulated and three downregulated (**Figure 4B**; **Supplementary Table 7**). The top five upregulated genes encode apolipoprotein B mRNA editing enzyme, catalytic polypeptide-like 3A (*APOBEC3Z1*), carbonic anhydrase 2 (*CA2*), inositol polyphosphate-4-phosphatase type II B (*INPP4B*)*, SRGAP3*, and the lncRNA ENSBTAG00000065240. The downregulated genes in cDC2 encoded C-X-C motif chemokine ligand 10 (*CXCL10*), AT-rich interaction domain 5B (*ARID5B*), and a different lncRNA (ENSBTAG00000066907).

**Figure 5:**
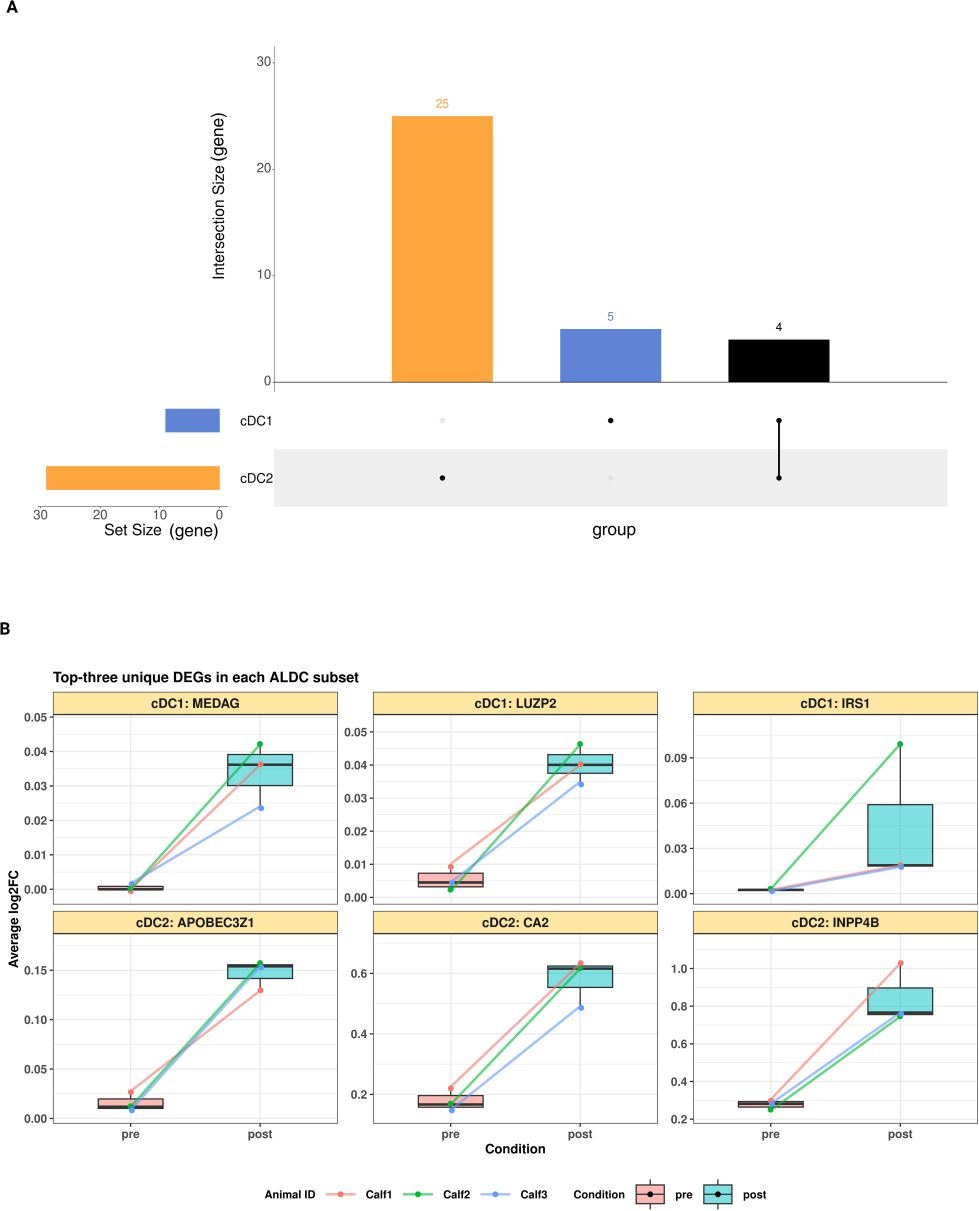
**(A)** Upset plot displaying the number of DEGs (adjusted P-value < 0.05) in each ALDC subset following BCG vaccination, with connected lines indicating shared DEGs between subsets. **(B)** The top-three unique DEGs following BCG vaccination in each ALDC subset are displayed with the expression level representing average log2 fold changes.

The sharing of DEGs among two ALDC subsets was visualised using an upset plot (**Figure 5A**). cDC2 had more unique DEGs (25) than cDC1 (5). Shared DEGs between cDC1 and cDC2 were *MSANTD1, SRGAP3, GAS8* and the lncRNA ENSBTAG00000065240. The top-three unique DEGs post-BCG in each ALDC subset are displayed in **Figure 5B**.

## 4 Discussion

BCG is the current gold standard vaccine against tuberculosis, yet the mechanisms behind its heterogenous protection require further study in DCs, which play a core role in antigen presentation and immune induction. Here, the major populations of ALDCs were identified as cDC1 and cDC2 subsets. cDC1 is recognised for its ability to cross-present antigens via MHC-I, thereby activating CD8+ T-cells. Conversely, cDC2 is thought to communicate via MHC-II to activate CD4+ T-cells. Thus, cDC1 and cDC2 may play distinct roles in immunity against bTB following BCG vaccination. Applying pseudo-bulk differential expression tests enabled us to capture the most consistent biological changes across animals and cell types, leading to high confidence in the expression differences reported between cDC1 and cDC2 in naïve calves, and in response to BCG. While we attempted gene set enrichment analyses on these DEGs, we did not identify any meaningful enrichments, potentially due to the small number of high-confidence DEGs available. Therefore, we focus our interpretations on the most notable DEGs in relation to existing literature. Our results not only offer single-cell transcriptomic profiles of bovine ALDCs, which have not been previously described, but also revealed genes responsive to BCG in ALDC subsets, improving understanding of how protective responses are generated by BCG vaccination.

### 4.1 Differentially expressed genes between cDC1 and cDC2 at homeostasis

**Figure 6:**
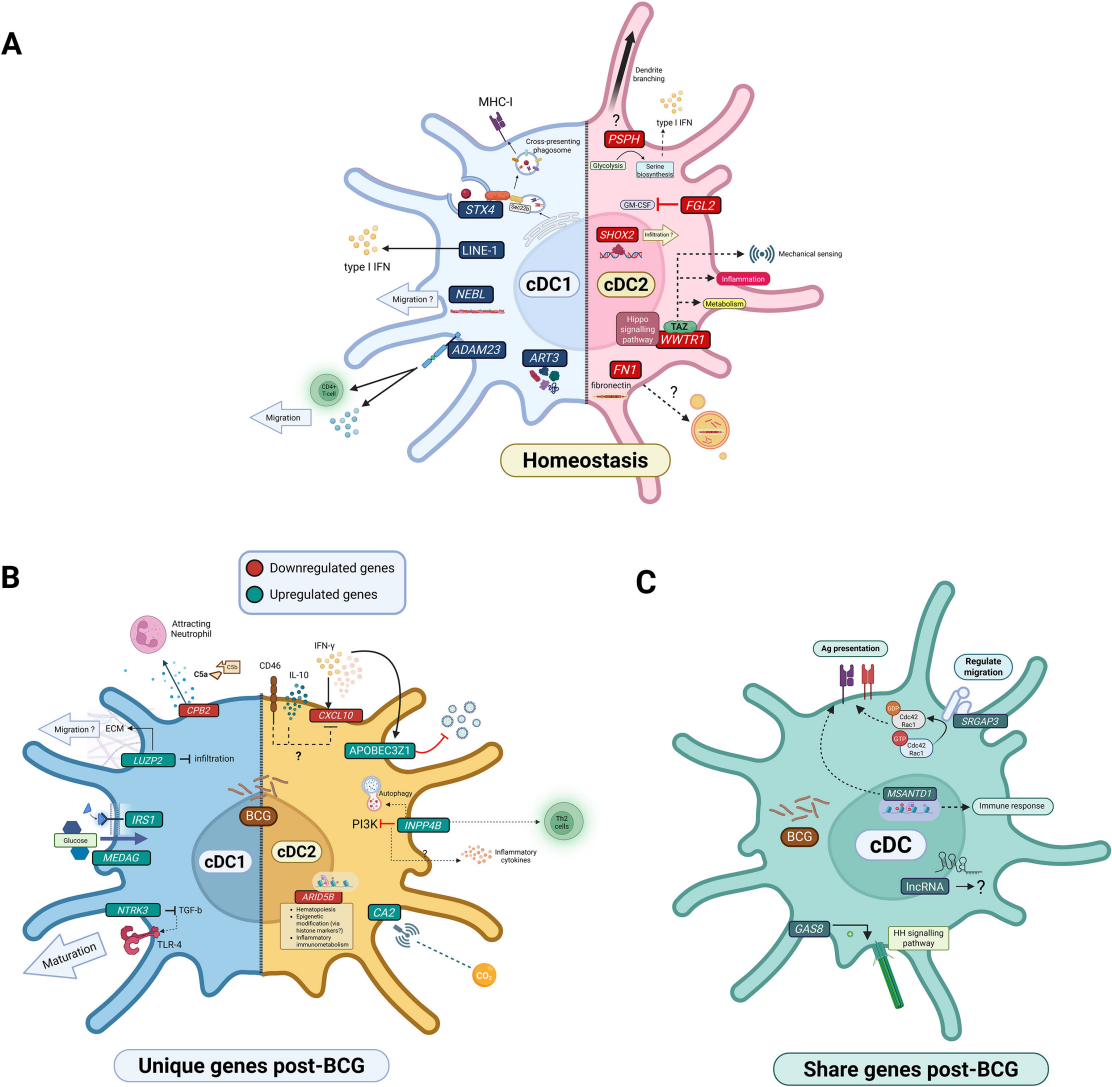
**(A)** Summary of upregulated DEGs distinguishing cDC1 and cDC2 at homeostasis. **(B)** Summary of cDC1 or cDC2 specific responses to BCG, with up/downregulated genes in green/red. **(C)** Summary of upregulated DEGs shared by cDC1 and cDC2 following BCG vaccination. The image was created using BioRender.com.

Comparison between cDC1 and cDC2 transcriptomic profiles in the pre-BCG samples identified subset-specific marker genes (**Figure 6A**). The upregulation of LINE-1 retrotransposable element ORF2 protein isoform in cDC1 may act to stimulate innate immunity via type I IFN (58, 59), which can enhance cross-presentation capability (60). *STX4*, also upregulated in cDC1, encodes syntaxin 4, a plasma membrane protein required in cross-presentation by interactions with Sec22b that serve to transform phagosomes into cross-presenting phagosomes (61). Nebulette, encoded by *NEBL* and upregulated in cDC1, belongs to the actin-binding nebulin protein family, which plays a role in cell migration (62). *ADAM23*, upregulated in cDC1, encodes an integrin receptor, which plays a role in antigen presentation, leading to CD4+ T-cell activation and cytokine production (63). Recently, *ADAM23* was discovered in pre-migratory cDCs, where its upregulation was proposed to promote extracellular matrix detachment and DC migration (64). Finally, *ART3*, upregulated in cDC1, has roles in post-translational modification, and was expressed in homeostatic human monocytes (65).

Regarding genes upregulated in cDC2, *FN1* was the top DEG, indicating potential fibronectin (Fn) production. Fn normally functions in cell adhesion, motility and differentiation (66); however, DC-derived Fn can enhance pathogen infectivity (67). This supports the hypothesis that Fn derived from cDC2 is linked to antigen presenting roles, supported by multiple studies. For example, exosomes derived from BCG/Mtb-infected macrophages can promote T-cell activation (68) and mycobacterial antigen affinity to Fn (69, 70). Phosphoserine phosphatase, encoded by *PSPH*, is a rate-limiting enzyme for L-serine formation – its upregulation in cDC2 indicates a shift from glycolysis to serine biosynthesis, representing a control mechanism for the type I IFN response in pDCs (71, 72). *FGL2*, encoding fibrinogen-like protein 2, suppresses DC differentiation by blocking granulocyte-macrophage colony-stimulating factor (GM-CSF) signalling (73). *SHOX2* has general roles in DNA binding and its expression was positively correlated with DC infiltration in multiple cancers (74). Lastly, *WWTR1* encodes transcriptional co-activator with PDZ-binding motif (TAZ) protein, which is a key molecule for the hippo signalling pathway (75). Taz has emerging roles in innate immune cells, including mechanical sensing and regulating DC metabolism (76, 77), promoting inflammation (78) and responses to LPS stimulation (79), which may relate to the inflammatory phenotype of cDC2.

Interestingly, a past scRNA-seq study on bovine migratory DCs in mesenteric lymph nodes reported many shared DEGs with our study (24). For instance, a cDC1 cluster showed 43 shared markers to our pre-BCG cDC1 population, including *CXCL9*, *TXN*, *BATF3*, and *STX4*. Similarly, the cDC2 cluster from the same study showed 31 markers shared with our pre-BCG cDC2 population, including *CCL17*, *FN1*, *PSPH*, and *SHOX2*. This similarity indicates bovine migratory DCs share functions beyond their anatomical compartment. Genes distinguishing cDC1 and cDC2 require further functional exploration, but already provide valuable subset-specific marker candidates for bovine ALDCs and perhaps overall migratory DCs.

### 4.2 Responses of cDC1 to BCG vaccination

cDC1 displayed subset-specific changes in the expression of genes consistent with metabolic remodelling (*MEDAG, IRS1*), maturation (*NTRK3*), migration (*LUZP2*), and lymphocyte recruitment activity (*CPB2*). The only downregulated gene post-BCG was *CPB2*, which has anti-inflammatory functions, acting through the complement system - this suggests an increase of activated C5a, which attracts and activates neutrophils (80), consistent with the neutrophil recruitment ability of cDC1 during bacterial infection (81).

*MEDAG*, upregulated post-BCG, is involved in cell differentiation and glucose uptake in adipocytes, promoting adipogenesis (82). While consistent with the known upregulation of lipid metabolism observed post-BCG (83), this gene has not previously been assigned roles in immune cells. Another metabolic gene, *IRS1*, encodes a key substrate for insulin receptor, involved in signal transduction and glucose transport by insulin (84), which indicates increased glycolytic activity induced by TLR ligation or the higher energy requirements of mature DCs (85). The upregulation of both genes indicates metabolic remodelling toward glycolysis in response to BCG, potentially an early signal of epigenetic and metabolic modifications (6, 86).

Another gene upregulated by cDC1 was *NTRK3*, encoding tropomyosin receptor kinase C (TrkC), which suppresses TGF-β, a factor that impedes DC maturation and downregulates TLR4. Thus, increased TrkC may enhance DC maturation and upregulate TLR4 (87, 88). *LUZP2* encodes leucine zipper protein 2, a prognostic biomarker in various human diseases (89). In the context of DCs, its functions in the extracellular matrix may influence migration velocity or haptotaxis (90) (**Figure 6B**).

### 4.3 Responses of cDC2 to BCG vaccination

A previous study found that BCG was preferentially uptaken and carried by SIRPα+ ALDCs (28), corresponding to cDC2 described here, which may explain the higher number of DEGs compared to cDC1. The top upregulated DEGs specific to cDC2 included *CA2*, encoding carbonic anhydrase 2, which governs intracellular pH and aids phagocytic immune cells in detecting inflammation (91). *CA2* upregulation thus suggests a role for cDC2 in sensing inflammation following BCG vaccination. *APOBEC3Z1* is induced by IFN stimulation in immature DCs (92) and is highly correlated with IFN-γ expression during antiviral activity (93). Its upregulation suggests the relationship between *APOBEC3Z1* and IFN-γ also exists in response to BCG. *INPP4B* is an inhibitor of PI3K signalling, suggesting a possible link to upregulation of inflammatory cytokine responses in DCs after TLR activation (94, 95).

Surprisingly, *CXCL10* which encodes an inflammatory cytokine that recruits lymphocytes to infection sites (96), showed downregulation in cDC2. This might have resulted from CD46 activation in DCs, which can occur after BCG exposure (97). Furthermore, DCs infected by Mycobacteria strains containing the *sigE* mutation can exhibit high IL-10 and low CXCL10 production (98). Thus, CD46 and IL-10 could display regulatory roles post-BCG (**Figure 6B**). Another gene downregulated in cDC2 after BCG vaccination, *ARID5B*, encodes a transcription factor that regulates B-cell growth and haematopoiesis, along with inflammatory responses and phagocytosis in LPS-stimulated monocytes (99). The role of *ARID5B* in DCs and its relationship with BCG thus warrants further investigation.

### 4.4 BCG responses shared between cDC1 and cDC2

Commonly upregulated genes between cDC1 and cDC2 were associated with transcriptional regulation (*MSANTD1*), DC migration and antigen presentation (*SRGAP3*), and cell signalling (*GAS8*) (**Figure 6C**). *MSANTD1* encodes a protein containing the SANT domain, which plays a role in transcriptional regulation and histone tail modifications (100). A transcriptomic study connected *MSANTD1* to immune responses and antigen presentation, revealing a negative correlation with activated DC infiltration (101). *SRGAP3* encodes a regulatory protein that inactivates members of Rho-GTPase protein family, specifically Cdc42 and Rac1, resulting in inhibition of cell spreading and adhesion (102). Furthermore, Cdc42 and Rac function in antigen uptake, where their downregulation during DC maturation indicates a shift from endocytosis toward antigen presentation (103). *GAS8* encodes dynein regulatory complex subunit 4; although rarely reported in connection with DCs, this gene has functions related to hedgehog signalling (Hh) and cilia function (104). Interestingly, Hh plays an important role in maturation and migration of monocyte-derived DCs (105), while the functions of *GAS8* in cilia may underlie essential roles in DCs, for example, DC maturation (106), developmental signalling pathways (107), and immune responses (108). The shared upregulation of lncRNA ENSBTAG00000065240 emphasises the potential unexplored roles of non-coding genes in the response to Mycobacteria (109).

## 5 Conclusions

By capturing single-cell transcriptomic profiles of bovine ALDCs we have identified ALDC subset-specific genes and expression responses to BCG vaccination. We offer transcriptomic evidence for metabolic remodelling, DC maturation, lymphocyte recruitment, and immune responses following BCG vaccination. Additionally, novel genes without previous connections to DCs were discovered that offer novel markers for BCG responses leading to protective immunity.

## Data Availability

The datasets presented in this study can be found in the Gene Expression Omnibus (GEO) repository (https://www.ncbi.nlm.nih.gov/geo/) GEO accession: GSE312782.
