# Supplementary material for: Single cell transcriptomics reveals dendritic cell subsets in bovine afferent lymph and immune cell-resolved responses to BCG vaccination
Source: Front Immunol. 2026 Apr 7;17:1764014. doi: 10.3389/fimmu.2026.1764014 (PMC13095522; doi:10.3389/fimmu.2026.1764014)
Supplement: Supplementary file 1 [file DataSheet1.docx]

Supplementary Material

#
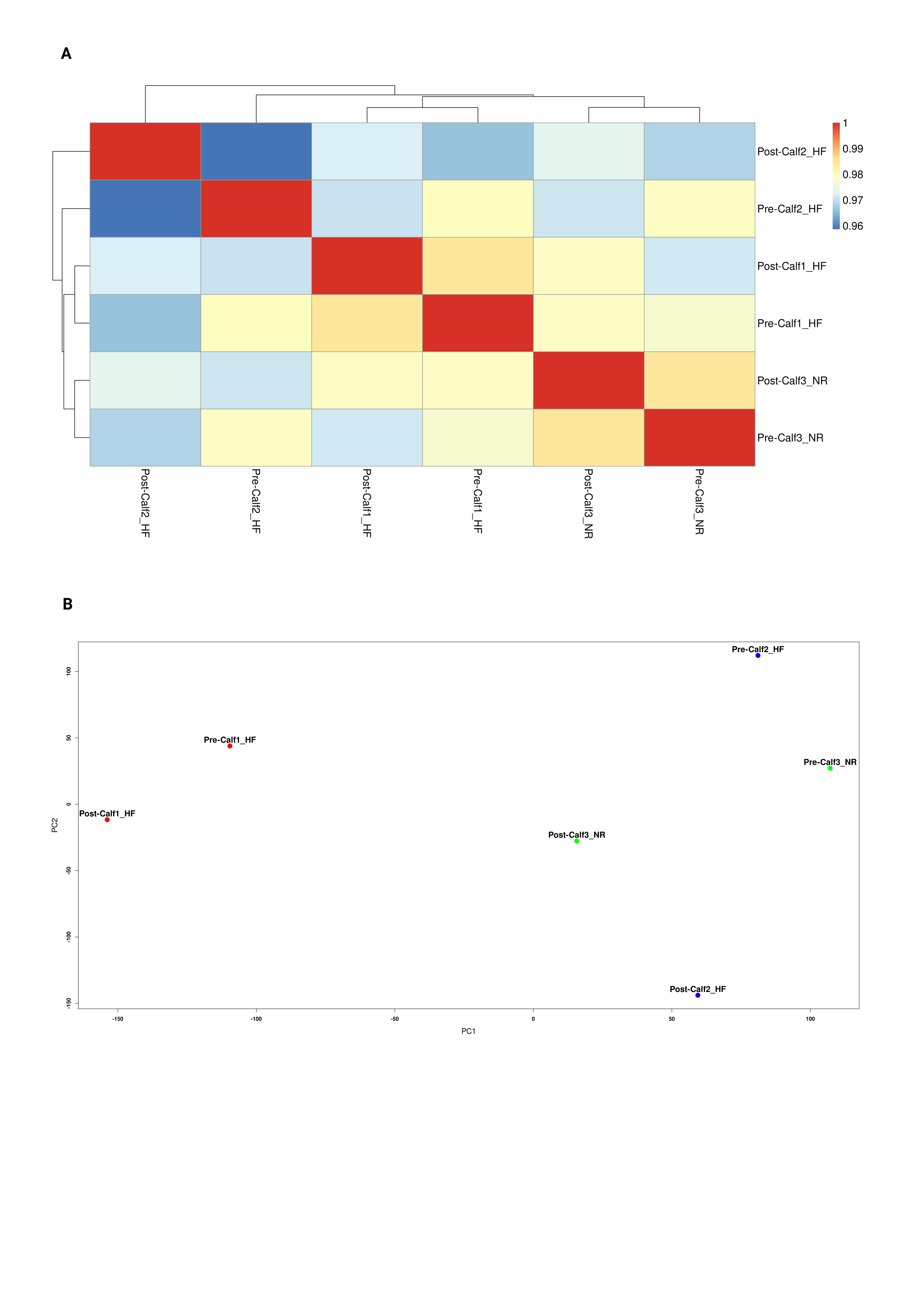
Supplementary Figures

**Supplementary Figure 1** (A) Heat map displays correlation of gene counts from all samples with high correlation (>0.958) indicating high reproducibility in all samples and no impact of breed (HF; Holstein-Friesian, NR; Norwegian Red). (B) Principal component analysis based on gene counts across all six samples.

#
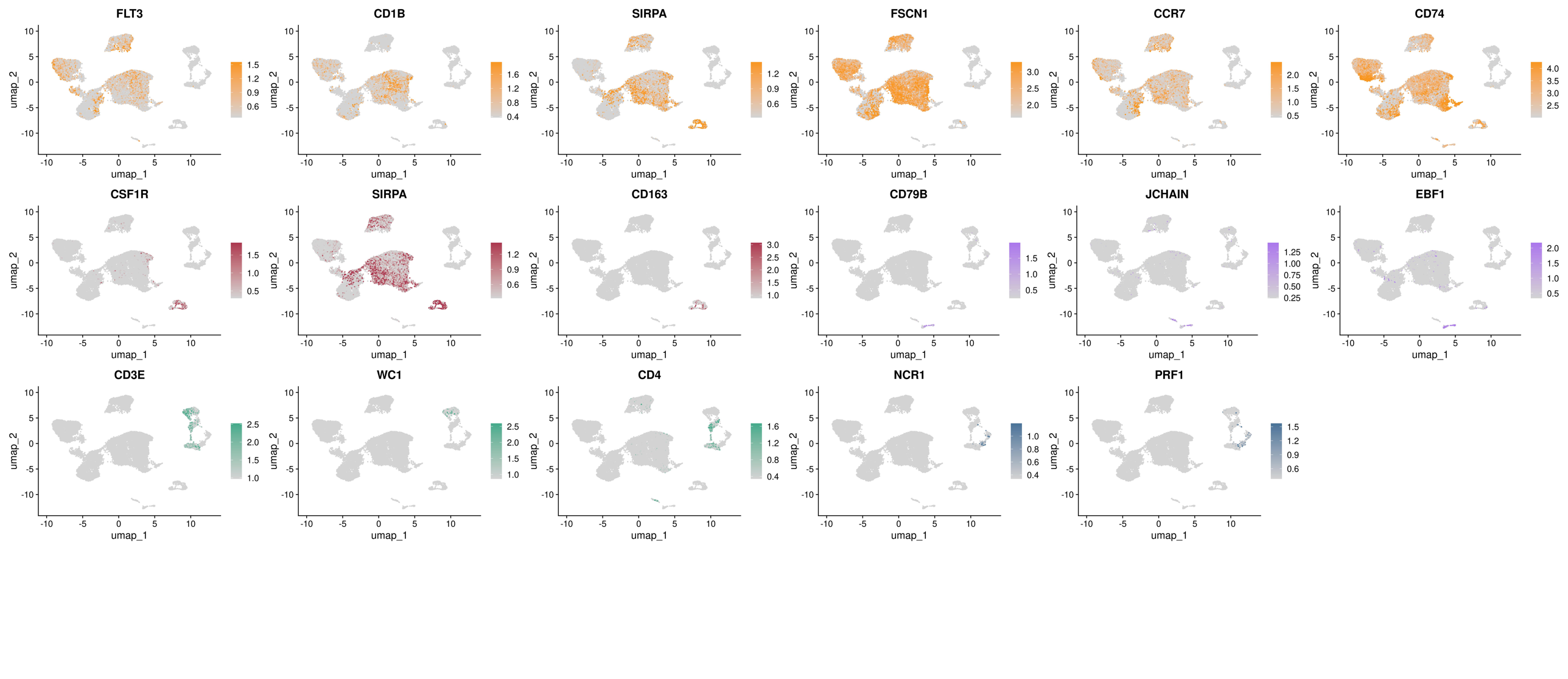


**Supplementary Figure 2** Feature plots showing the expression of *a priori* defined marker genes in DCs (orange), monocytes (red), B-cells (purple), T-cells (green), and NK cells (blue).


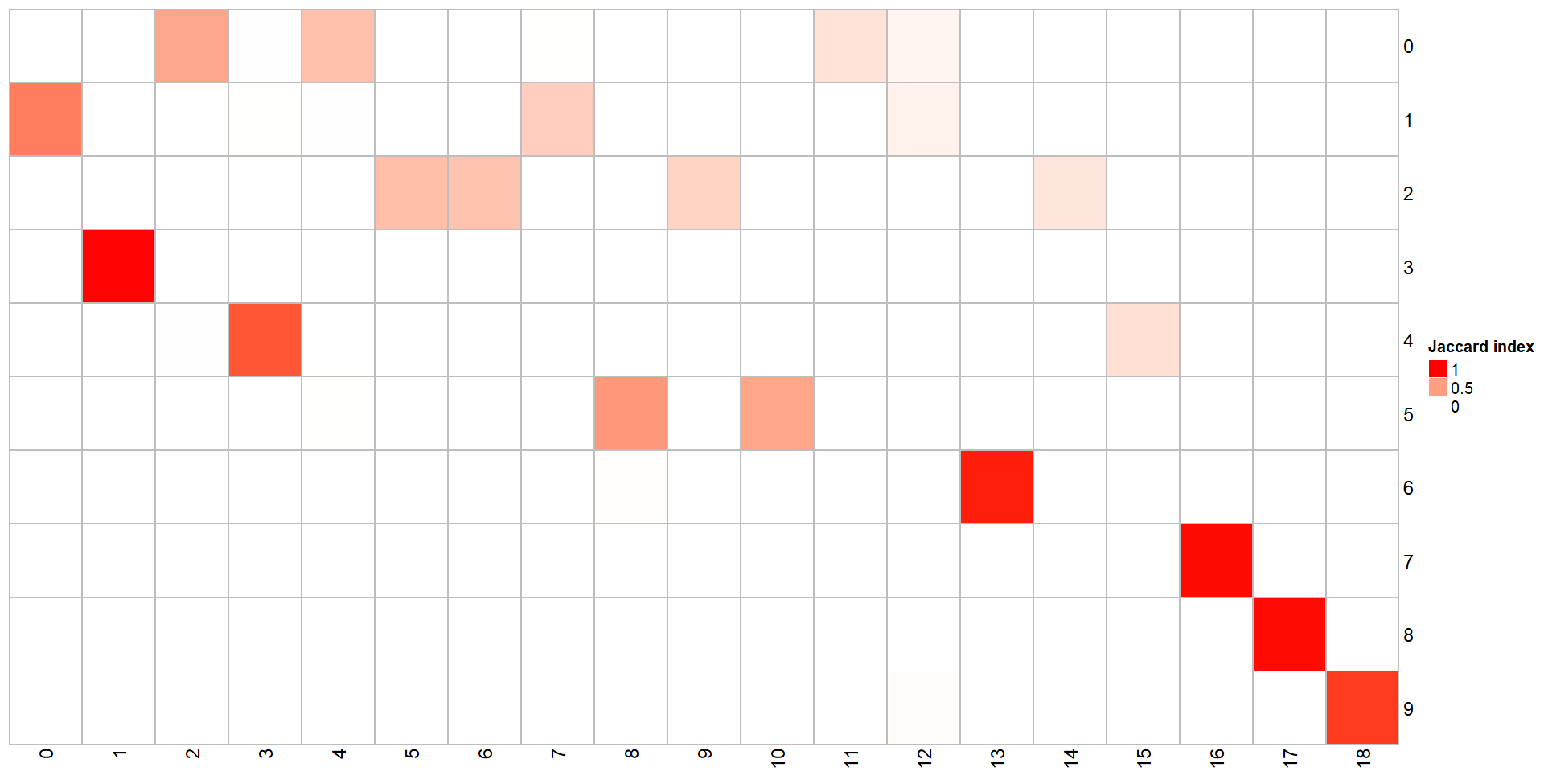


**Supplementary Figure 3A** The heatmap comparison between resolution 0.4 (Y-axis, 10 clusters) and resolution 1.2 (X-axis, 19 clusters), showed high correlation (expressing in red colour) in various clusters.


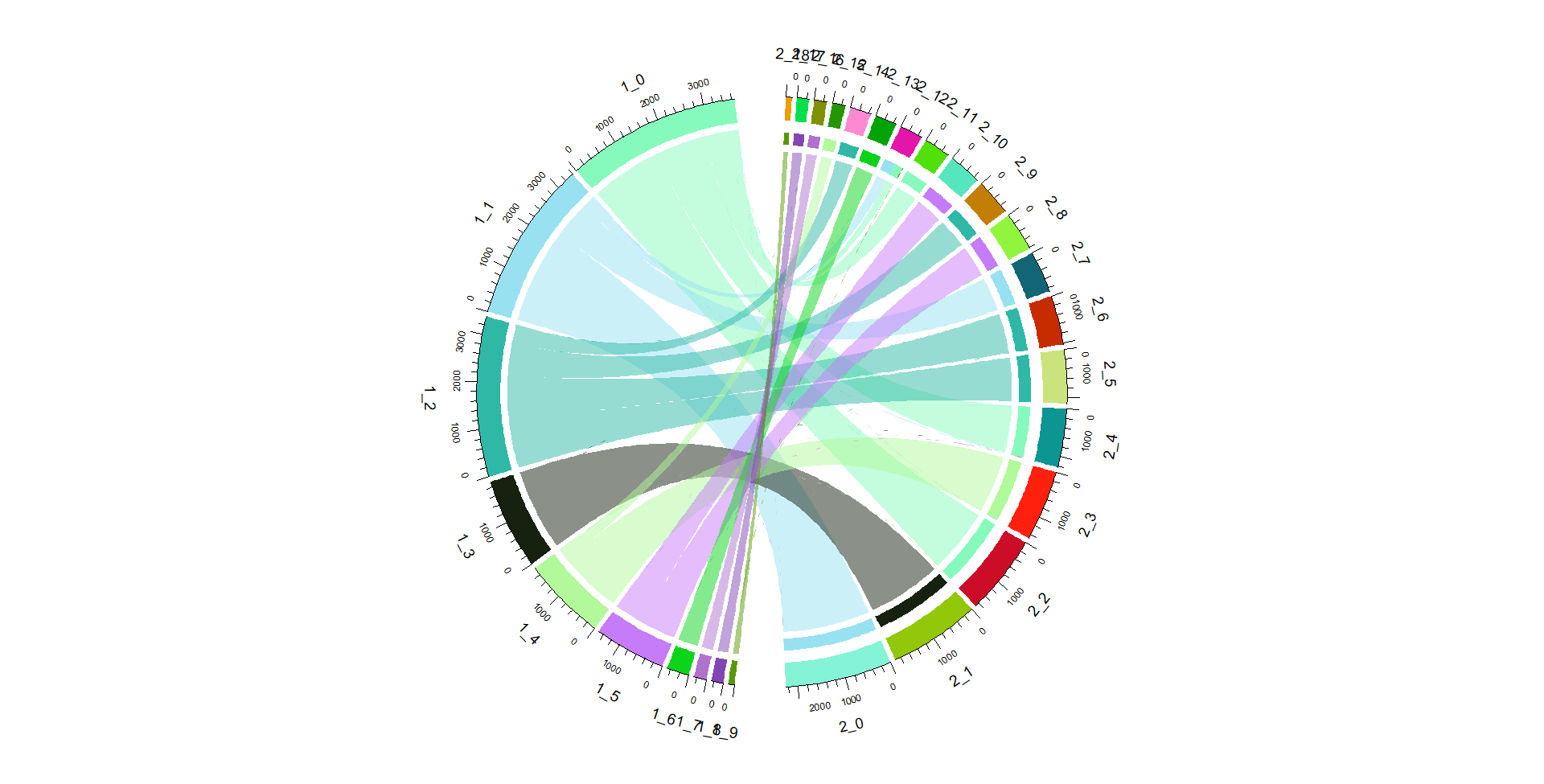


**Supplementary Figure 3B** A chord plot illustrates the transition of population branching from lower resolution (left) to higher resolution (right).


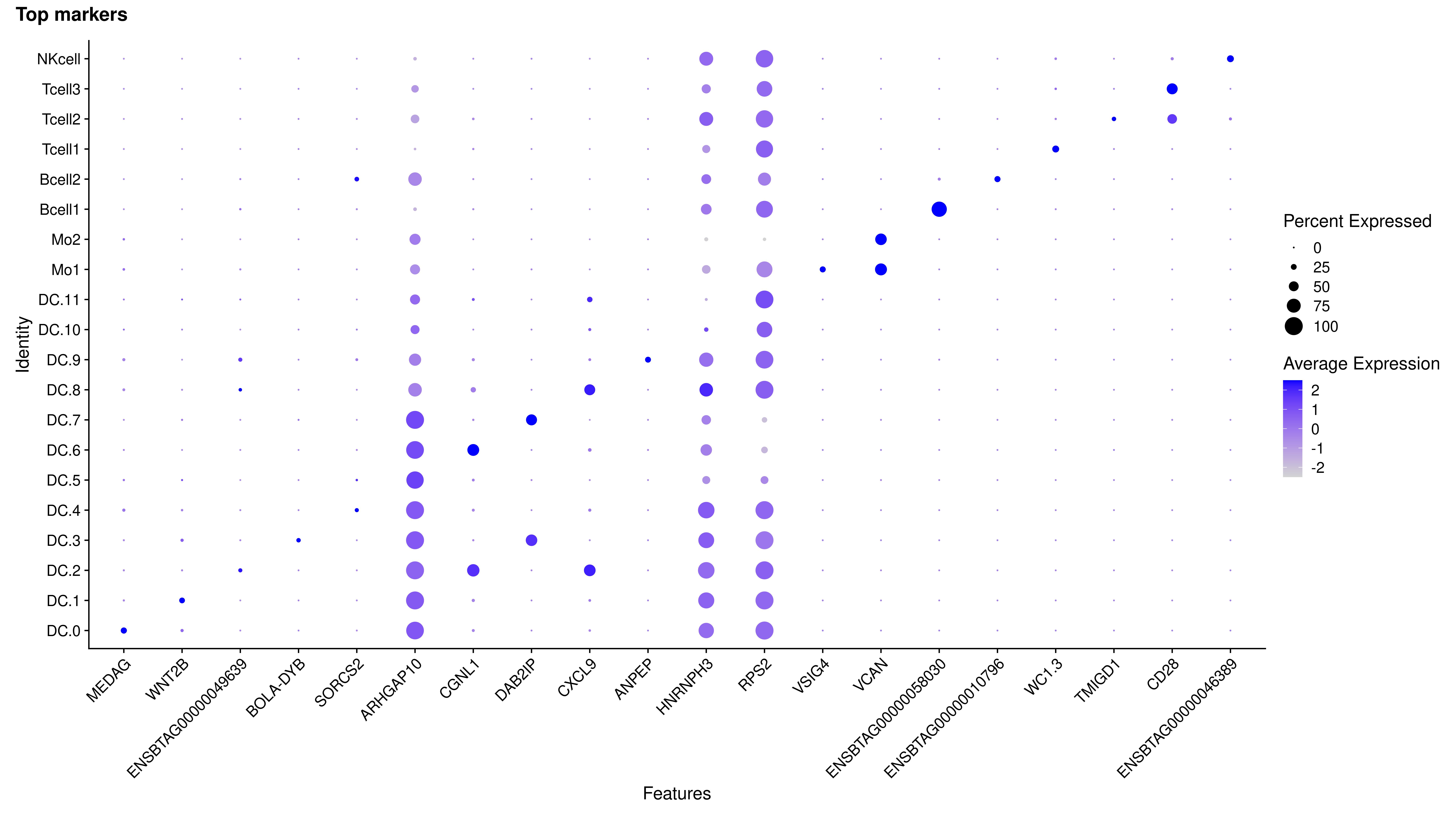


**Supplementary Figure 4** Dot plot displays top marker genes from individual cluster combined all cell populations from both conditions.


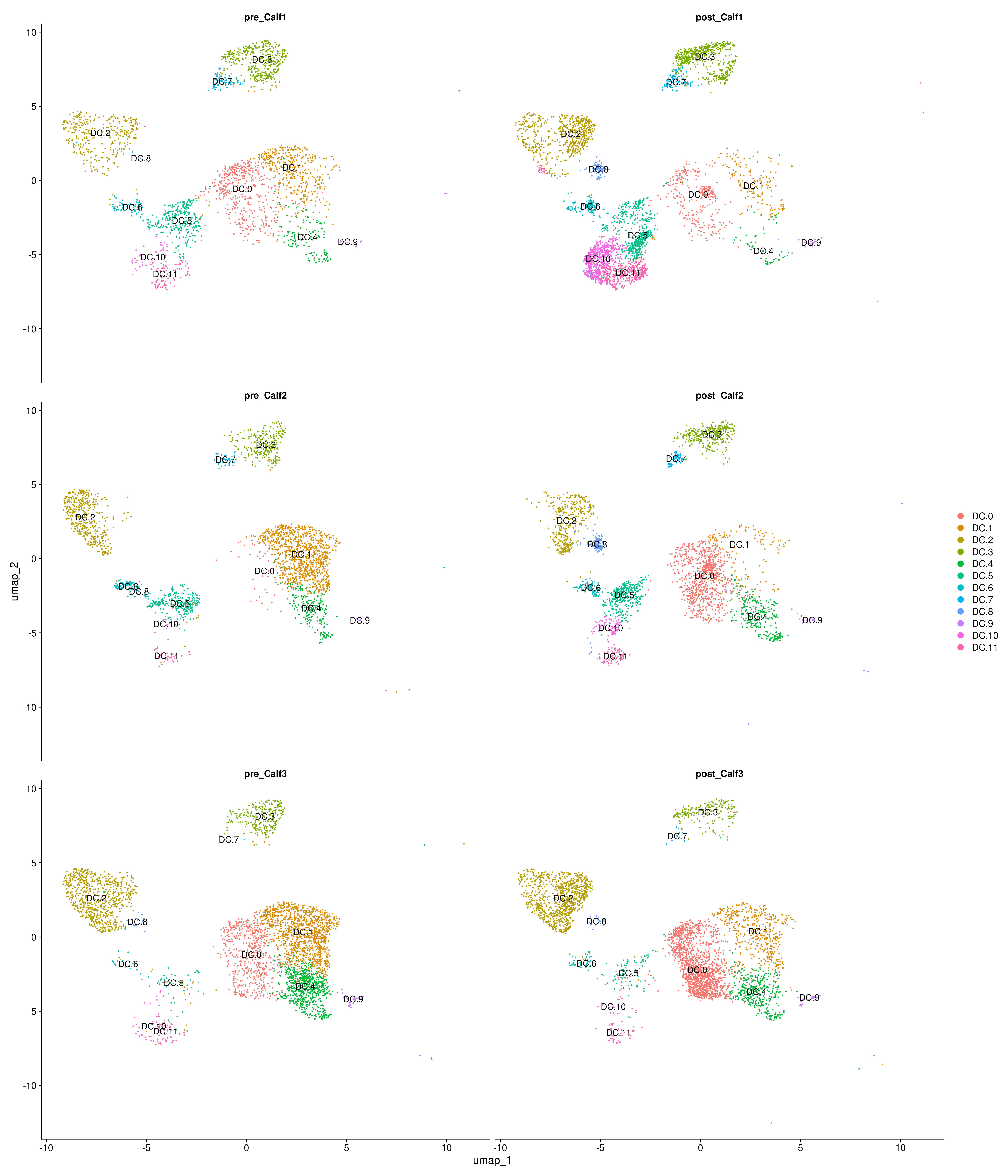


**Supplementary Figure 5** The UMAP displays ALDC clusters, each divided by animals and experimental conditions (pre- and post-BCG).


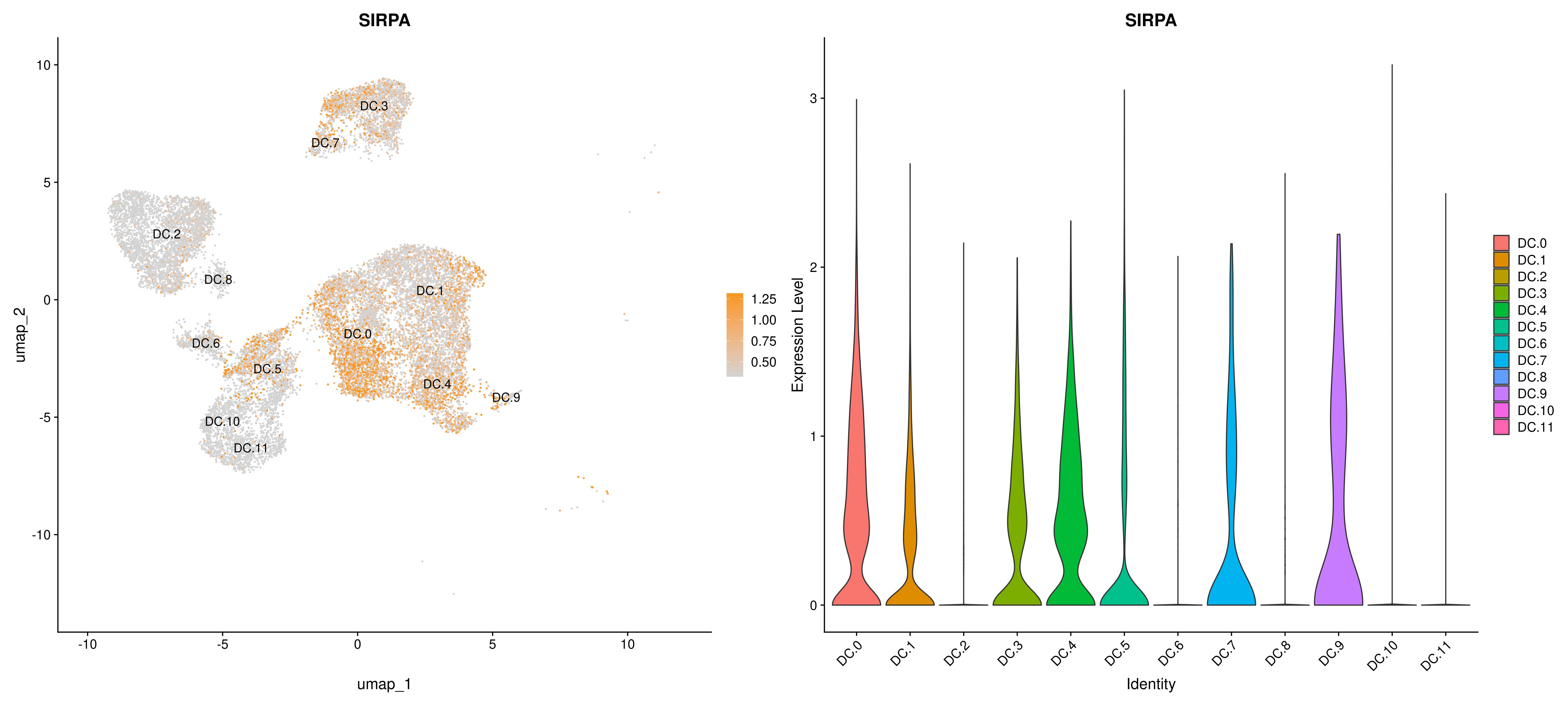


**Supplementary Figure 6** Feature plot and violin plot show expression level of *SIRPA*, used as marker gene for cDC2 identification.


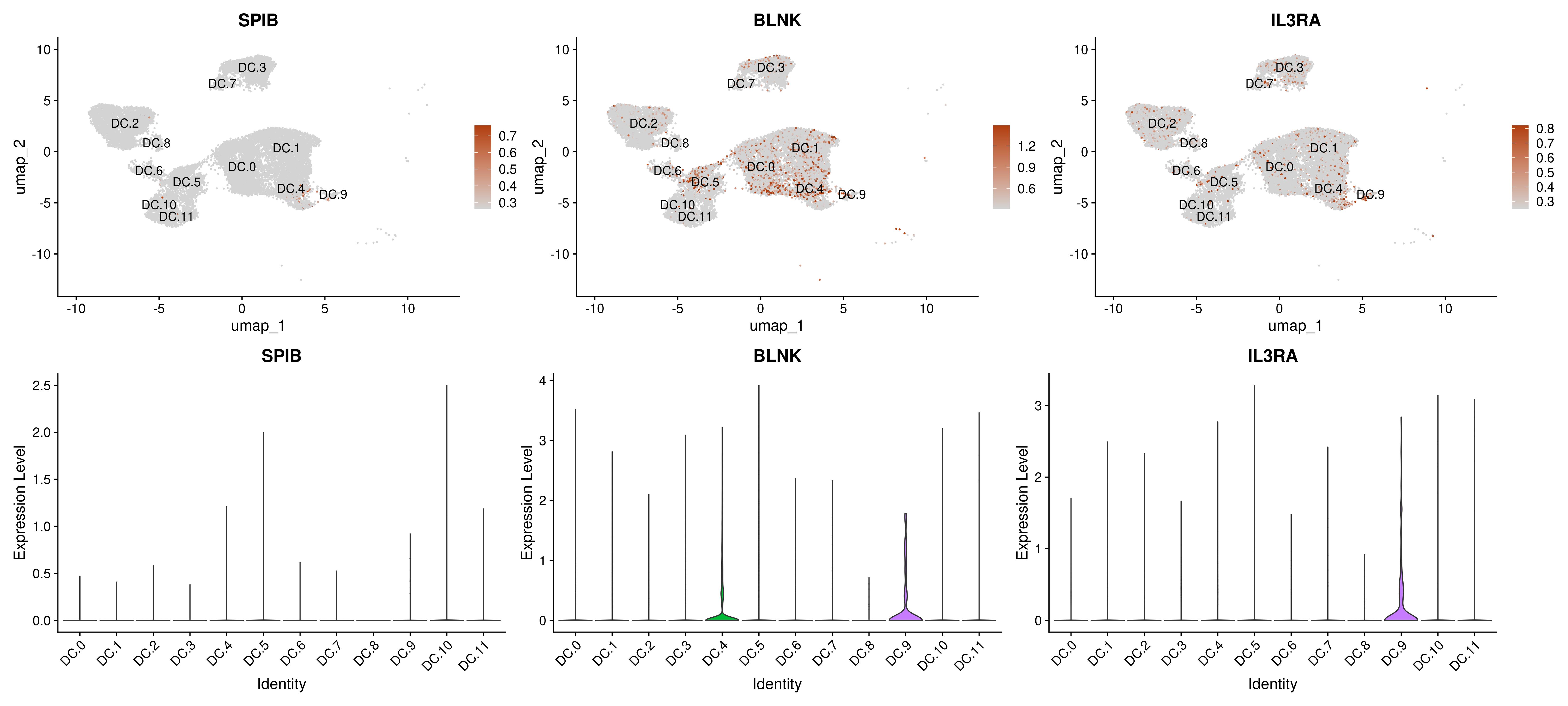


**Supplementary Figure 7** Feature plot and violin plot show expression level of *SPIB, BLNK,* and *IL3RA*, used as marker genes for putative tDC identification.


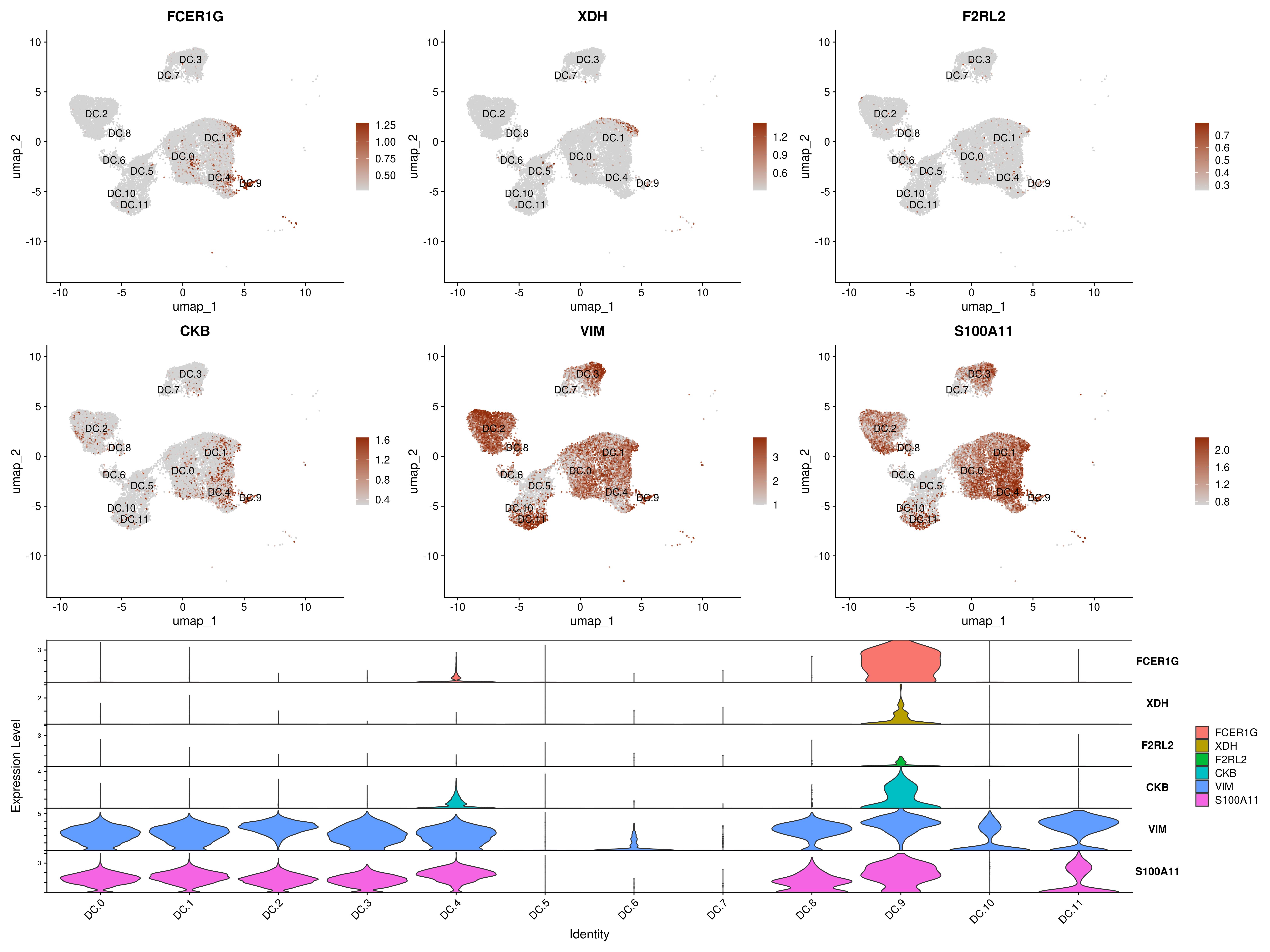
**Supplementary Figure 8** Feature plot and violin plot show expression level of *FCER1G, XDH, F2RL2, CKB, VIM,* and *S100A11,* used as marker genes for putative DC3 identification.


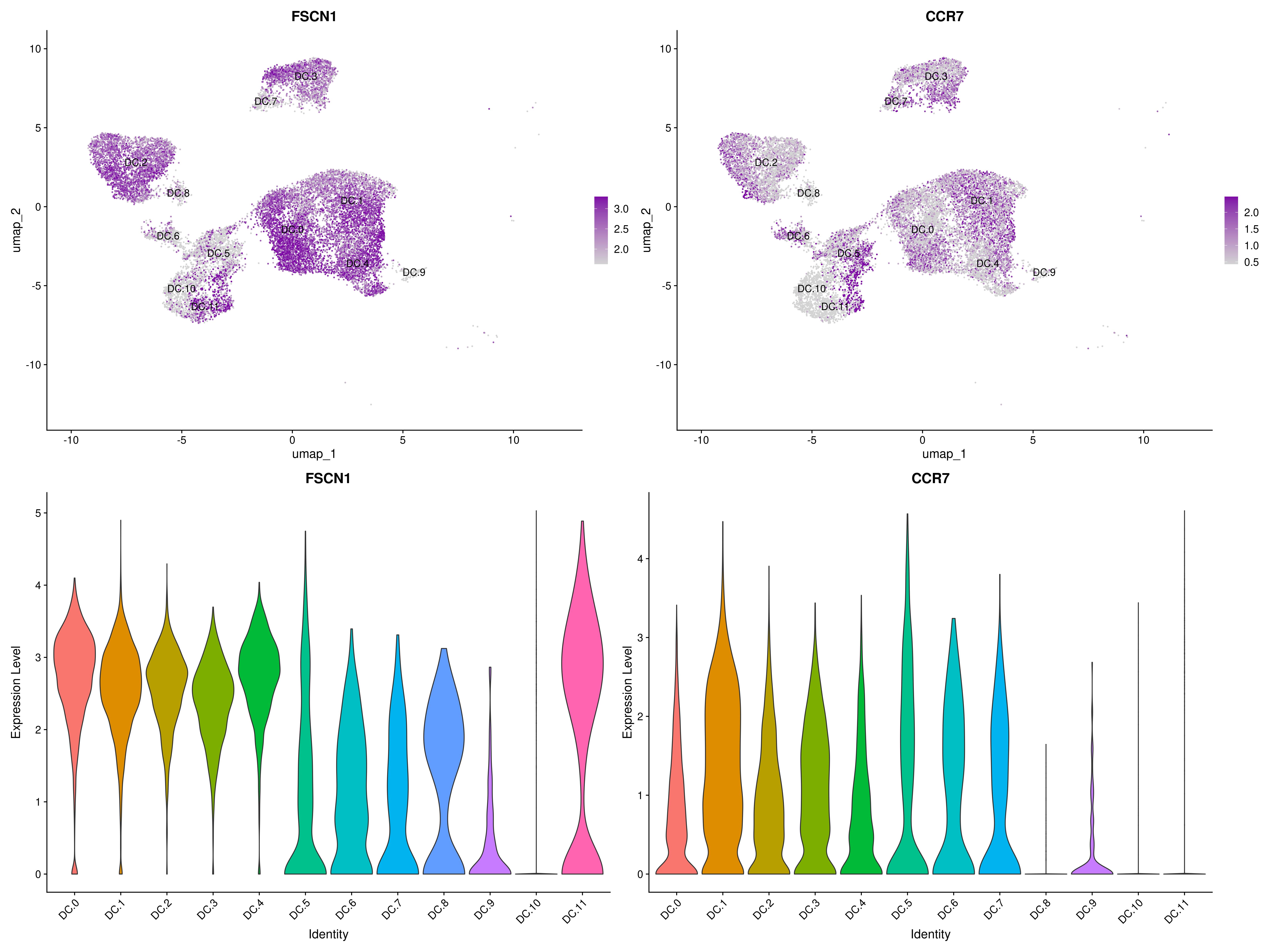


**Supplementary Figure 9** Feature plot and violin plot show expression level of *FSCN1* and *CCR7,* used as marker genes for migratory profile identification.


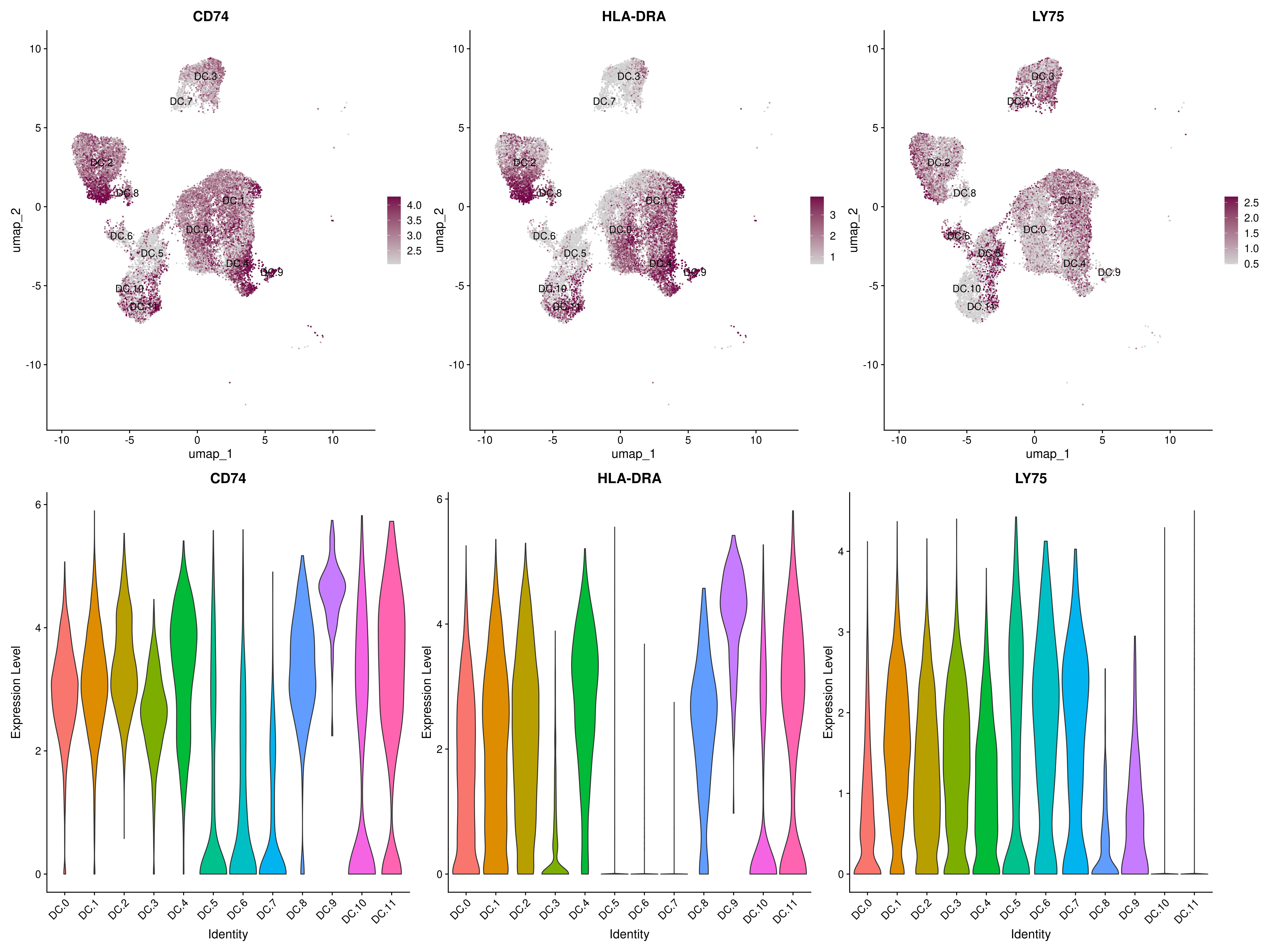


**Supplementary Figure 10** Feature plot and violin plot show expression level of *CD74, HLA-DRA,* and *LY75* used as marker genes for identifying antigen presentation function.


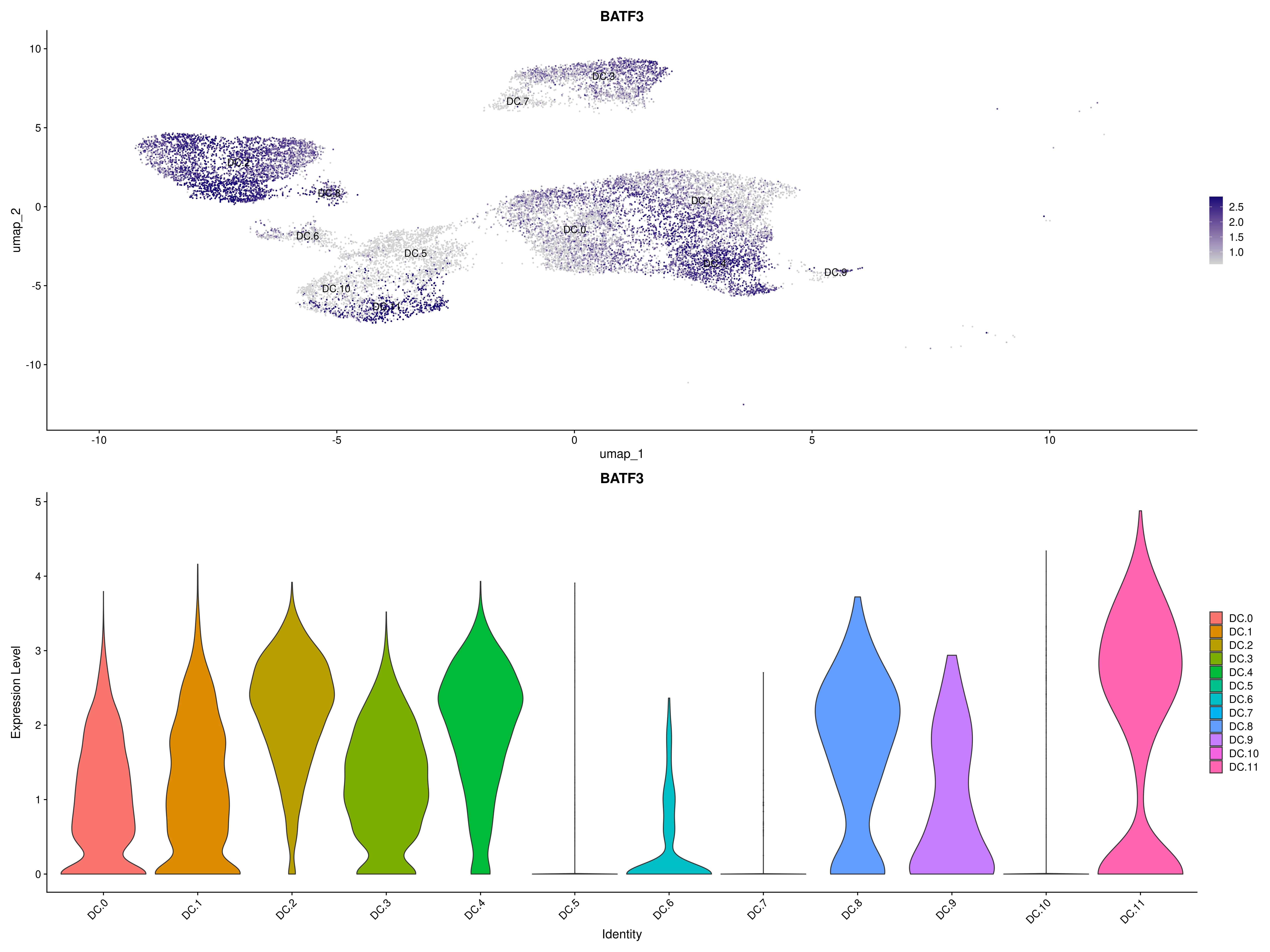


**Supplementary Figure 11** Feature plot and violin plot show expression level of *BATF3* across cDC1 and cDC2 populations.
